# Enrichment of Autotrophic Denitrifiers From Anaerobic Sludge Using Sulfurous Electron Donors

**DOI:** 10.3389/fmicb.2021.678323

**Published:** 2021-06-07

**Authors:** M. F. Carboni, A. P. Florentino, R. B. Costa, X. Zhan, P. N. L. Lens

**Affiliations:** ^1^Department of Microbiology, School of Natural Sciences and Ryan Institute, National University of Ireland Galway, Galway, Ireland; ^2^Department of Biochemistry and Organic Chemistry, Institute of Chemistry, São Paulo State University, Araraquara, Brazil; ^3^Department of Civil Engineering, School of Engineering, College of Science and Engineering, National University of Ireland Galway, Galway, Ireland

**Keywords:** nitrogen removal, reduced sulfur compounds, pyrite, microbial diversity, enrichment

## Abstract

This study compared the rates and microbial community development in batch bioassays on autotrophic denitrification using elemental sulfur (S^0^), pyrite (FeS_2_), thiosulfate (S_2_O_3_^2–^), and sulfide (S^2–^) as electron donor. The performance of two inocula was compared: digested sludge (DS) from a wastewater treatment plant of a dairy industry and anaerobic granular sludge (GS) from a UASB reactor treating dairy wastewater. All electron donors supported the development of a microbial community with predominance of autotrophic denitrifiers during the enrichments, except for sulfide. For the first time, pyrite revealed to be a suitable substrate for the growth of autotrophic denitrifiers developing a microbial community with predominance of the genera *Thiobacillus*, *Thioprofundum*, and *Ignavibacterium*. Thiosulfate gave the highest denitrification rates removing 10.94 mM NO_3_^–^ day^–1^ and 8.98 mM NO_3_^–^ day^–1^ by DS and GS, respectively. This was 1.5 and 6 times faster than elemental sulfur and pyrite, respectively. Despite the highest denitrification rates observed in thiosulfate-fed enrichments, an evaluation of the most relevant parameters for a technological application revealed elemental sulfur as the best electron donor for autotrophic denitrification with a total cost of 0.38 € per m^3^ of wastewater treated.

## Graphical Abstract

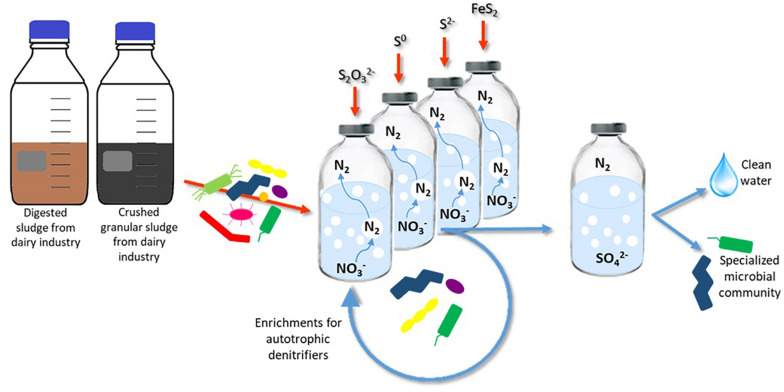

## Introduction

Nitrate pollution in water bodies is a global environmental issue, threatening natural ecosystems, e.g., causing eutrophication and algal blooms, as well as threatening human life, e.g., causing methemoglobinemia and cancer (Liu et al., 2009; Sutton et al., 2011; Sun and Nemati, 2012). Several anthropogenic activities lead to nitrate pollution in the environment, such as the use of nitrogen-based fertilizers in agriculture, use of nitrogen-based chemicals in industry, and the uncontrolled discharge of wastewaters in water bodies (Glass and Silverstein, 1999; Almasri and Kaluarachchi, 2004; Yang et al., 2018). Numerous physical methods have been used to treat nitrate-contaminated wastewater, such as ion exchange, reverse osmosis, adsorption, and electrodialysis (Shrimali and Singh, 2001). However, the high costs of these techniques limit their applicability (Ghafari et al., 2008; Pu et al., 2015). Alternatively, biological denitrification processes have been developed as cost-effective technologies, achieving high removal efficiencies and low energy consumption (Shrimali and Singh, 2001).

Both autotrophic and heterotrophic denitrifying bacteria are able to convert nitrate to dinitrogen in the presence of inorganic (CO_2_ or HCO_3_^–^) or organic carbon as energy source, respectively (Cui et al., 2019). Heterotrophic denitrification is the most widely used biological process applied for nitrate removal in wastewater containing high concentrations of organic carbon and ammonia (Kostrytsia et al., 2018). Nevertheless, in many contaminated waters, organic carbon is absent or present in too low concentrations to guarantee total denitrification. For this reason, they require a supplemental external carbon source, such as methanol, ethanol, acetic acid, glycerol, or sugars (Shrimali and Singh, 2001). This addition represents an increase in the operational costs and can result in a secondary organic pollution due to the possible generation of by-products during the treatment (Zhang et al., 2015).

As an alternative treatment process, autotrophic denitrification has been widely studied in the last decades (Moon et al., 2008; Shao et al., 2010; Zhou et al., 2011; Wood and Wood, 2014). The most common studied electron donors for autotrophic denitrification are hydrogen (Vasiliadou et al., 2009) and reduced sulfur compounds (RSCs), such as elemental sulfur (S^0^), sulfide (S^2–^), and thiosulfate (S_2_O_3_^2–^) (Manconi et al., 2007; Torrentó et al., 2011; Di Capua et al., 2016). The use of inorganic compounds as electron donor results in decreasing costs, since they are often available as pollutant in the wastewater and thus simultaneous denitrification and decontamination can be achieved (Tong et al., 2017). The choice of the electron donor greatly influences the denitrification activity and microbial community as different microorganisms have different abilities to metabolize the supplied electron donor. All the so far reported autotrophic denitrifiers belong to the *Proteobacteria* phylum (Di Capua et al., 2019). *Parococcus* is commonly reported to use hydrogen as electron donor, while *Thiobacillus* and *Sulfurimonas* are known to use RSCs. Hydrogen is mostly applied for denitrification in drinking water (Lee and Rittmann, 2003; Wang and Qu, 2003), but its high cost and handling safety requirements hamper its use in full-scale plants. Many RSCs are environmental pollutants, since they are highly toxic and corrosive. However, they are converted to a harmless compound (sulfate) during autotrophic denitrification. For this reason, the use of RSCs as electron donor for denitrification has attracted interest in the last few decades.

Recently, different types of non-common inorganic electron donors that are naturally present in groundwater have been studied: ferrous iron (Fe^2+^), pyrrhotite (FeS), and pyrite (FeS_2_) are suitable substrates for *Thiobacillus denitrificans* (Straub et al., 1996; Schippers and Jørgensen, 2002; Bosch et al., 2012). Interestingly, pyrite, an abundant mineral on the Earth’s crust with very low solubilization in water, complies an important role in natural attenuation of nitrate (Jørgensen et al., 2009). Jørgensen et al. (2009) studied denitrification in an aquifer in Denmark and demonstrated that pyrite contributes for 65–80% of the denitrification in the anoxic zone. Different studies have been conducted to improve the denitrifying performance using pyrite as electron donor (Pu et al., 2015; Kong et al., 2016). However, to date, pyrite-oxidizing microbial communities capable of reducing NO_3_^–^ or NO_2_^–^ have not yet been characterized.

This study investigated four different electron donors (FeS_2_, S_2_O_3_^2–^, S^0^, and S^2–^) in batch incubations, for which denitrification occurs according to the following stoichiometric reactions (1–4) (Thauer et al., 1977; Cardoso et al., 2006; Bosch et al., 2012):

(1) FeS2+3NO3+-2H2O→Fe(OH)3+1.5N2+2SO42-+H+ΔG0,-782.3[kJ/reaction]

(2) $${\text{S}}_{2}{\text{O}}_{3}^{2-}+1.6{\mathrm{NO}}_{3}^{-}+0.2H_{2}\text{O}\to 2SO_{4}^{2-}+0.8N_{2}+{\text{H}}^{+}\Delta {\text{G}}^{0,}-765.7\left[\mathrm{kJ}/\mathrm{reaction}\right]$$

(3) 5S0+6NO3+-2H2O→5SO42-+3N2+4H+ΔG0,-547.6[kJ/reaction]

(4) 5HS-+8NO3+-3H+→5SO42-+4N2+4H2OΔG0,-743.9[kJ/reaction]

This study aimed (i) to enrich autotrophic sulfur-oxidizing denitrifying microorganisms with either FeS_2_, S_2_O_3_^2–^, S^0^, or S^2–^ as electron donor at mesophilic conditions using anaerobic digested sludge and crushed anaerobic granular sludge as the inoculum, (ii) to analyze how the microbial community develops and changes depending on the substrate, starting from these two inocula, and (iii) to comparatively evaluate the denitrification performance of the tested electron donors for their benefits/drawback from an applied point of view. The employed strategy allowed the selection, for the first time, of a community of autotrophic denitrifiers grown directly on pyrite. Moreover, an investigation to define the best electron donor for the autotrophic denitrification is proposed, gathering the most relevant information such as microbial development, denitrification rates, and cost analysis, revealing elemental sulfur to be the most suitable electron donor, among those tested, for the autotrophic denitrification process.

## Materials and Methods

### Source of Biomass

Two anaerobic sludges were used as inoculum to enrich the autotrophic denitrifying communities: granular sludge (GS) from a dairy wastewater treatment plant collected from a UASB reactor operating at ambient temperature in Kilconnell (Ireland) (Castilla-Archilla et al., 2020), and digested sludge (DS) from the wastewater treatment plant of the dairy industry Dairygold Co-Operative Society (Mitchelstown, Ireland) (Dessì et al., 2020). Total solids (TS) were 0.030 and 0.023 g TS.g sludge^–1^ in DS and GS, respectively. Volatile solids (VS) were 0.023 g TS.g sludge^–1^ in DS and 0.016 g TS.g sludge^–1^ in GS. Granular sludge was crushed in a MB800 blender (Kinematica, Lucerne, Switzerland) for 5 min.

### Electron Donors

Four different sulfur compounds were tested as electron donor and energy source (Table 1) to perform autotrophic denitrification from synthetic wastewater: FeS_2_, S^0^, S_2_O_3_^2–^, and S^2–^. FeS_2_ (99.8% grade, 325 mesh diameter from Sigma-Aldrich, St. Louis, United States), chemically synthetized S^0^ (99.5% grade, 100 mesh diameter, from Fischer Scientific, Hampton, United States), or thiosulfate as Na_2_S_2_O_3_ (99% grade from Fischer Scientific, Hampton, United States) was added in excess of the stoichiometric molar ratio (Eqs. 1–3) to always ensure the presence of the electron donor for the bioconversion. The molar ratio was 1.6, 4.8, and 12.1 times higher than the stoichiometric value for, respectively, thiosulfate, elemental sulfur, and pyrite. The solid substrates elemental sulfur and pyrite also provided a solid surface as support for the cell growth. Due to toxicity of sulfide on autotrophic denitrifiers, 3 mM of Na_2_S⋅9H_2_O (98% grade from Fischer Scientific, Hampton, United States) was added to the enrichments.

**TABLE 1:** Concentration of electron donors in each batch bottle and molar ratio of e**^–^** donor/e**^–^** acceptor.

Electron donor	Concentration [mM]	Molar ratio e^–^ donor/e^–^ acceptor
Thiosulfate	20	1
Sulfide	3	0.15
Elemental Sulfur	80	4
Pyrite	80	4

### Experimental Setup

Enrichment of autotrophic denitrifiers from the anaerobic sludges in a synthetic wastewater was performed in 250-ml serum bottles with a working volume of 125 ml. The synthetic wastewater was designed to ensure that all the minimal requirements for anaerobic bacterial growth were met. Nitrate was added as KNO_3_ to a final concentration of 20 mM NO_3_^–^ L^–1^. The sterile anoxic basal medium was prepared as described by Stams et al. (1993) and was composed of (g L^–1^): 0.41 KH_2_PO_4_, 0.53 Na_2_HPO_4_⋅2H_2_O, 0.3 NH_4_Cl, 0.3 NaCl, and 0.1 MgCl_2_⋅6H_2_O; 1 ml L^–1^ of each acid and alkaline trace elements solution; 0.1 g L^–1^ yeast extract (Alfa Aesar, Ward Hill, United States); and 1 ml L^–1^ resazurin sodium salt solution (Fisher Scientific, Hampton, United States). 0.11 g L^–1^ CaCl_2_⋅2H_2_O, 4 g L^–1^ NaHCO_3_, and 0.2 ml L^–1^ vitamin solutions were added filter-sterilized to the autoclaved medium. The pH of the medium was kept in the range of 7–7.5. Serum bottles filled with anoxic medium were sealed with butyl rubber stoppers (Ochs Laborbedarf, Bovenden, Germany) and aluminum crimp caps. The headspace of the bottles was flushed with pure argon to a final pressure of 1.5 atm.

The enrichments were performed in triplicate. Controls without sludge or electron acceptor were analyzed in triplicate as well to investigate, respectively, any abiotic activity and the contribution of endogenous organic carbon present in the sludge to the denitrification process. Bottles were statically incubated at 30°C. Enrichments containing pyrite and sulfide as electron donor were kept in the dark to avoid iron and sulfide photo-oxidation.

Due to their physical nature, the enrichment for the soluble thiosulfate (ThEnr) and sulfide (S^2–^Enr) and the solid elemental sulfur (S^0^Enr) and pyrite (PyEnr) electron donors followed different protocols (Supplementary Figure 1). In all cases, the first set of bottles was inoculated with 20% (v/v) of each of the two inocula. Afterward, for the soluble substrates, 20% (v/v) of the previous cultures was transferred whenever nitrate and nitrite were completely depleted or when the denitrification activity reached the stationary phase. For the solid electron donors, the particles were transferred to fresh medium after centrifugation at 4000 rpm for 3 min (Eppendorf, Hamburg, Germany), followed by washing steps in phosphate buffer (pH 7). Moreover, also for PyEnr and S^0^Enr, 20% (v/v) of the previous set of bottles was transferred in case the bacteria were also growing in suspension and not only attached to the surface of the particles. An exception was made for S^0^Enr, for which the denitrification activity was decreasing along the first four transfers (from 100% nitrogen removal to 65% in the 4th transfer): the cultures were left 19 extra days inside the bottles between the 4th and 5th transfer (from day 36 to 55) to allow for a better bacterial attachment to the sulfur particles. An enrichment was considered concluded when the denitrification rates of two consequential transfers varied less than 5%. The number of transfers of each incubation with the associated total duration is shown in Table 2.

**TABLE 2:** Number of transfers and total duration of the enrichments for every electron donor and inoculum.

Incubation	No. of transfers	Total duration of incubation [days]
DS ThEnr	5	22
GS ThEnr	5	20
DS PyEnr	5	108
GS PyEnr	5	108
DS S^0^Enr	6	71
GS S^0^Enr	6	74
DS S^2–^Enr	4	34
GS S^2–^Enr	4	34

### DNA Extraction

DNA was extracted in duplicate samples from the inoculum sources and from the early stationary phase of each of the triplicate enrichments after the 6th transfer for elemental sulfur and the 5th transfer for the other electron donors. Samples for DNA extraction were not taken from the sulfide incubations since the denitrification activity stopped after the 3rd transfer, suggesting that the community of autotrophic denitrifiers was not active. DNA was extracted from 2 ml for the sludges and 10 ml for the enrichment using the Qiagen DNeasy Power Soil extraction kit (QIAGEN, Hilden, Germany) according to the manufacturer’s protocol. Purity and concentration of the extracted DNA were analyzed using a NanoDrop 2000 (ThermoFisher, Waltham, United States). Extracted DNA samples were kept at −20°C prior to sequencing.

### Microbial Community Analysis

Sequencing was performed at Eurofins Genomics (Ebersberg, Germany). The total number of input sequences was 1,675,387, which was reduced to 1,619,992 after chimera detection and filtering based on the *de novo* algorithm of UCHIME (Edgar et al., 2011). The high-quality reads were processed using minimum entropy decomposition (Eren et al., 2013, 2015), which provided partition marker gene datasets into OTUs (operational taxonomic units). The taxonomic assignments were performed using the QIIME software package (version 1.9.1) using NCBI_nt as reference database. Abundances of bacterial taxonomic units were normalized using lineage-specific copy numbers of the relevant marker genes used to improve estimates of the relative abundance (RA) (Angly et al., 2014). The processed Illumina Miseq reads were deposited in the Sequence Read Archive of NCBI under accession number PRJNA707573.

### Analytical Methods

Samples were taken daily for the thiosulfate and elemental sulfur enrichments and weekly for the pyrite and sulfide enrichments. Samples were centrifuged at 13,200 rpm for 5 min in an Eppendorf AG MiniSpin 5452 centrifuge (Eppendorf, Hamburg, Germany) and filtered through a 0.22-μm membrane filter. Nitrite, nitrate, thiosulfate, and sulfate concentrations were determined by ion chromatography (Dionex Aquion, Thermo Scientific, United States) with an IonPac AS14A 4 × 250 mm column coupled with an AG14A 4 × 50 mm guard column and sodium carbonate 3.03 mM/sodium bicarbonate 0.97 mM eluent at a flow rate of 1 ml min^–1^ (Florentino et al., 2020). The ammonia concentration was determined using a Gallery Plus discrete analyzer (Thermo Scientific, United States) as described by Castilla-Archilla et al. (2020). Total organic carbon (TOC) measurements were carried out by a TOC analyzer (TOC-L, Shimadzu, Japan). The sulfide concentration was determined by the photometric method using methylene blue as previously described by Cline (1969) using a Shimadzu UV-1900 spectrophotometer (Shimadzu, Japan). The chemical structure of the solid particles recovered at the end of the pyrite and elemental sulfur enrichments was examined by Fourier transform infrared (FTIR) measurements on an attenuated total reflection accessory (ATR) (ATR-Nicolet iS5, Thermo Scientific) in the range from 4000 to 525 cm^–1^ with a 4 cm^–1^ resolution (Majzlan et al., 2011). Measurements of pH were acquired using a Mettler Toledo FiveEasy^TM^ (FP20, US). Total solids and Volatile solids were determined following the Standard Methods (APHA, 1998).

## Results

### Autotrophic Denitrification Activity of the Enrichments

**FIGURE 1:**
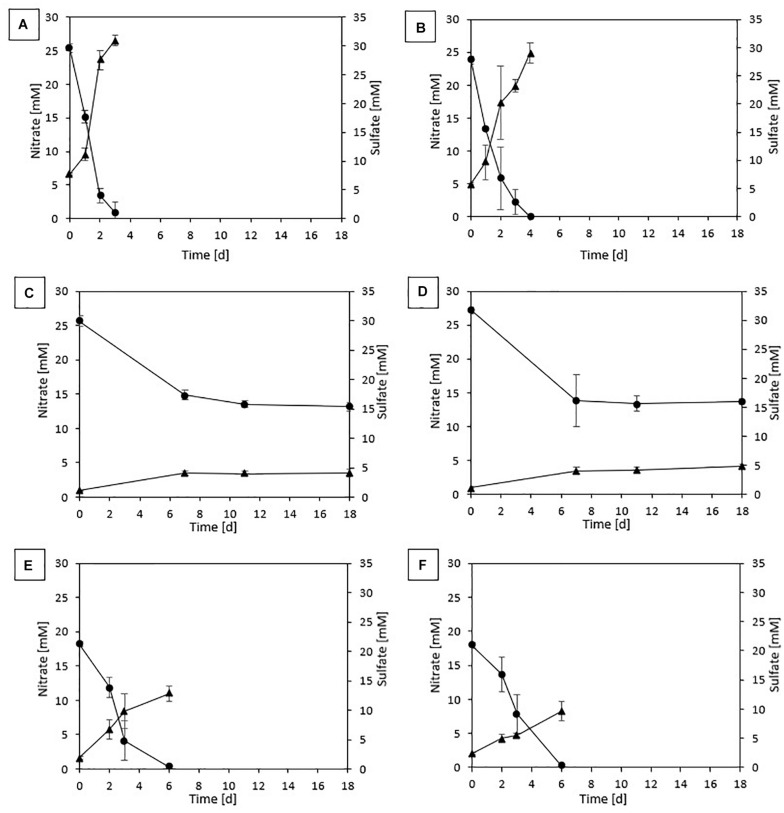
Profile of NO_3_^–^ (•) and SO_4_^2–^ (▲) of the last transfer in **(A)** ThEnr with DS, **(B)** ThEnr with GS, **(C)** PyEnr with DS, **(D)** PyEnr with GS, **(E)** S^0^Enr with DS, and **(F)** S^0^Enr with GS.

In the first incubation for both sludges, complete denitrification was achieved in 6 and 4 days with, respectively, thiosulfate and elemental sulfur as the electron donor. On the other hand, sulfide-driven denitrification reached a NO_3_^–^ removal efficiency of 85.3 (± 0.007)% for DS in 8 days and 93.9 (± 0.04)% for GS in 7 days. In the batches with pyrite as electron donor, 76.1 (± 0.04)% denitrification was achieved for DS and 89.5 (± 0.08)% for GS after 27 days of incubation. No lag phase was observed as NO_3_^–^ reduction immediately started with all tested electron donors for both sludges (Figure 1).

The presence and accumulation of nitrite during the enrichment were detectable only in the first transfer for thiosulfate, pyrite, and sulfide, reaching a maximum concentration of 2.37 mM on day 3 for the sulfide experiment with DS. In contrast, nitrite was found in every transfer for S^0^Enr with its accumulation between the 3rd and 5th transfer reaching 1.71 mM for DS and 2.07 mM for GS.

Figure 1 shows nitrate consumption and sulfate production profiles obtained in DS and GS incubations in the last transfer of the enrichments using thiosulfate (Figures 1A,B), pyrite (Figures 1C,D), and elemental sulfur (Figures 1E,F). NO_3_^–^ removal was observed for all electron donors and sludges tested. Regardless of the sludge used, thiosulfate-fed enrichments gave the highest denitrification rates (Table 3), being 1.4 and 1.5 times faster than elemental sulfur-driven denitrification for DS and GS, respectively, and 7.1 and 4.7 times faster than pyrite-driven denitrification for DS and GS, respectively. The denitrification efficiency reached 100% in the thiosulfate and elemental sulfur incubations on 4 and 6 days, respectively, while in the case of pyrite, it reached 51% for DS in 18 days (even if the variation in the last 7 days was only 1.5%) and 50% for GS in 11 days as a result of low denitrification kinetics.

**TABLE 3:** Denitrification rates (mM NO_3_^–^ day^–1^) for the four electron donors with the inocula (DS and GS) and the enriched cultures in the last transfer (DS_enr and GS_enr).

Sludge	Elemental sulfur	Thiosulfate	Pyrite	Sulfide
DS	7.65 ± 0.56	2.66 ± 0.35	1.10 ± 0.17	6.07 ± 0.05
DS_enr	7.78 ± 2.97	10.94 ± 0.28	1.54 ± 0.02	-
GS	21.55 ± 1.69	2.35 ± 0.36	1.68 ± 0.06	2.45 ± 0.47
GS_enr	5.83 ± 0.60	8.98 ± 1.79	1.90 ± 0.42	-

Regardless of the starting sludge, the nitrate removal efficiency for pyrite-driven denitrification decreased from the first to the last transfer. It is important to take into account that a wide variety of heterotrophic denitrifiers was active in the inocula contributing to the NO_3_^–^ removal using endogenous organic matter as electron donor, while in the last (5th) transfer, all the total organic carbon was consumed—and mainly autotrophic denitrifiers developed in the enrichment. In the controls carried out in the absence of electron donor, the inocula alone were able to reduce 42 and 61% of the supplied nitrate for DS and GS, respectively, while in the last three transfers, 6.4 (± 0.93) mM NO_3_^–^ was reduced by the DS enrichment corresponding to 25.4 (± 0.03)% removal of the total nitrate supplied to DS, whereas the GS enrichment reduced 5.9 (± 1.09) mM nitrate, corresponding to a 24.7 (± 0.03)% nitrate removal efficiency.

Data on the sulfide incubations are not reported as denitrification activity was no longer detected from the 4th transfer. In the 2nd and 3rd transfers of S2-Enr, up to 94 and 97% nitrate removal efficiency was reached for DS and GS biomass, respectively. At the end of the 2nd transfer, all the sulfide introduced in the bottles was consumed in the DS incubation and 93% in the GS enrichment. In the 3rd transfer, however, only 81 and 64% of sulfide was used during the incubation by the DS and GS enrichments, respectively.

### Denitrification Rates

Denitrification rates were calculated as the slopes of the curves describing the highest nitrate reduction during the first (inoculum sludges DS and GS) and last (enrichments DS_enr and GS_enr) transfer for each electron donor. A clear improvement in denitrification rates for thiosulfate-driven denitrification can be observed, being 4.11 and 3.82 times faster in the DS and GS enrichments, respectively (Table 3). Enrichments on pyrite and DS S^0^Enr lead to denitrification rates between 1.02 and 1.4 times faster, whereas the GS S^0^Enr slowed down the denitrification rate 3.7 times.

### Sulfate Production

**FIGURE 2:**
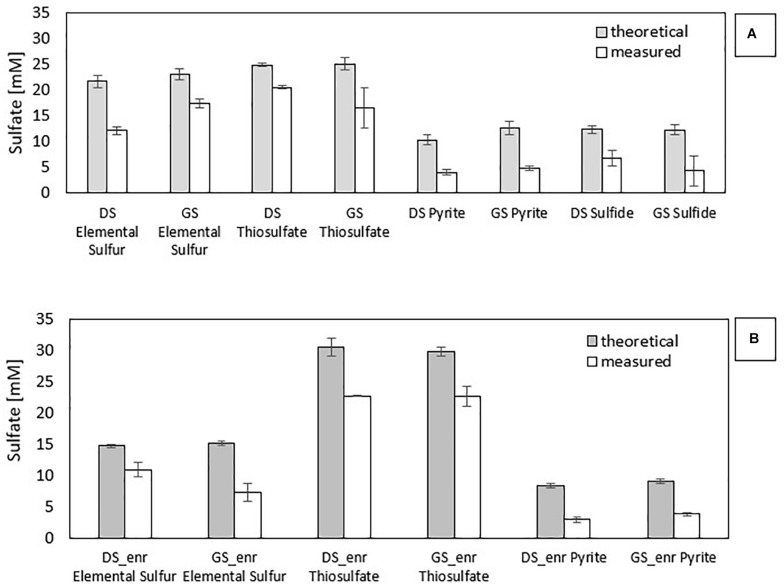
Comparison of measured and theoretical sulfate concentrations for all enrichments: **(A)** first incubation with the inocula and **(B)** last transfer with the enrichments.

Stoichiometrically, the reduction of 1 mol of nitrate leads to the production of 0.67 mol of sulfate with pyrite as electron donor (Eq. 1), 1.25 mol with thiosulfate (Eq. 2), 0.83 mol with elemental sulfur (Eq. 3), and 0.625 mol with sulfide (Eq. 4). Figure 2 shows the gap between the sulfate measured and sulfate theoretically expected for all electron donors investigated in the first (a) and last (b) transfer. The difference between them in the first transfer is due to the co-occurrence of heterotrophic (that does not produce sulfate) and autotrophic denitrification. This gap is also present in the last transfer, in which the nitrate removal activity was mostly due to autotrophic denitrifiers.

Figure 2B clearly shows that PyEnr has the highest gap since the measured sulfate concentration is only 36 and 42% of the amount expected from the theoretical calculation for PyEnr in the last transfer of DS and GS, respectively. In the elemental sulfur incubations, this difference in the concentrations occurred mainly in the last transfer of the enrichment with GS [GS_enr (48%)], while it is less for the last transfer of the enrichment with DS [DS_enr (74%)]. In thiosulfate incubations, instead, the two values are closer: 74 and 76% for DS_enr and GS_enr, respectively.

### Microbial Community Composition

**FIGURE 3:**
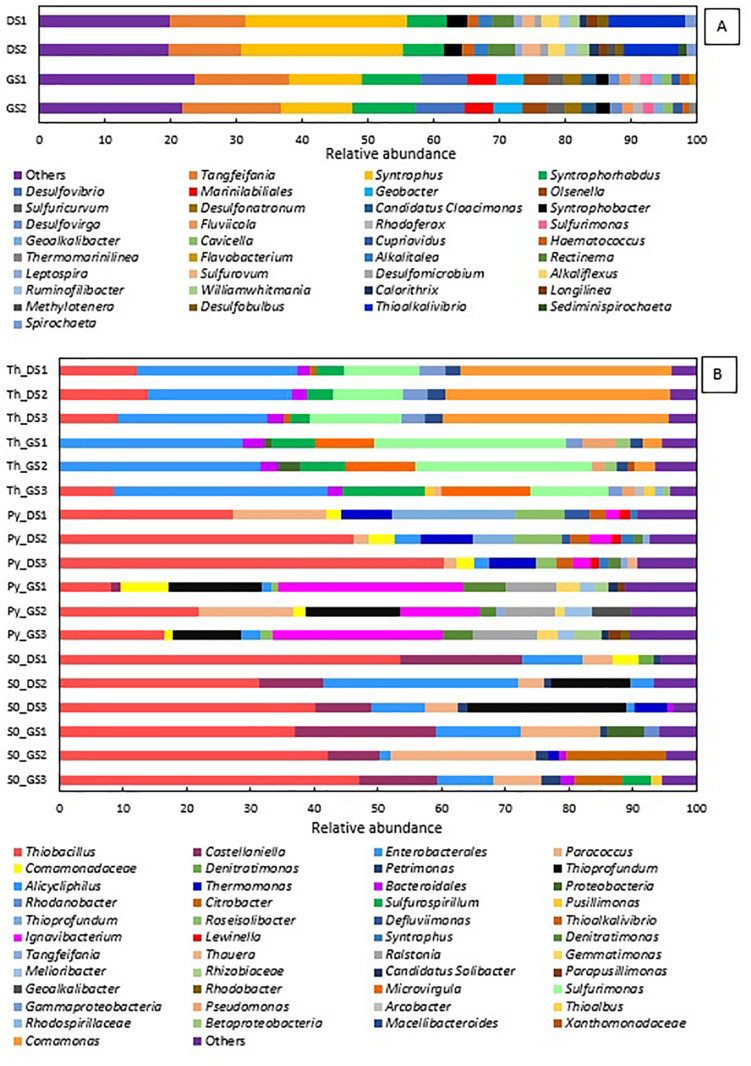
Relative abundance at genus level present in the **(A)** inocula—in duplicate—and **(B)** in the last transfer of ThEnr, PyEnr, and S^0^Enr—in triplicate.

Figure 3 shows how the microbial community shifts at the genus level from the two starting sludges (Figure 3A) to the enrichments for all electron donors investigated (Figure 3B). The microbial community diversity significantly reduced along the enrichments, implying an increased abundance of specialized groups, as several genera of denitrifiers became predominant in the cultures.

In the elemental sulfur and thiosulfate-fed enrichments, the phylum of *Proteobacteria* was always present in a RA between 94 and 98%, while in the pyrite-fed enrichments, it was detected between 60 and 87% RA. The genus *Thiobacillus* is present in almost all final enrichments with a predominant RA of 41.7 (± 9.2)% in the elemental sulfur incubations DS_enr, 42.0 (± 4.1)% in GS_enr, and 44.6 (± 13.6)% in the pyrite incubations in DS_enr. In the other analyzed conditions, this genus is present only in minor RA. In the two replicates of the thiosulfate incubations GS_enr, the genus *Thiobacillus* was below the detection limit. It is important to notice that the *Thiobacillus* genus was below the detection limit in the inoculum, but the belonging order was detected in a RA of only 0.25% in GS and 1.6% in DS. Except for *Thiobacillus* and the unclassified group of *Enterobacteriales*, the other genera developed in a very specific way depending on the electron donor applied.

The genera *Sulfurimonas*, *Comamonas*, and *Sulfurospirillum* got enriched in the thiosulfate incubations to 12.4, 34.6, and 3.9% in DS_enr, respectively, and to 23.5, 2.1, and 8.9% in GS_enr, respectively. The *Microvirgula* genus was found only in the thiosulfate incubations GS_enr with a RA of 11.3%. These genera were not identified at the genus level in the inoculum sludge, except for *Sulfurimonas* that was found in DS with a RA of 1.7%.

The same genera were identified in the pyrite enrichments, but with quite different RA: both enrichments developed *Thiobacillus*, 44.6% in DS_enr, and 15.5% in GS_enr. *Thioprofundus* and *Ignavibacterium* were also found in the pyrite incubations with, respectively, 8.5 and 2.78% RA in DS_enr and 13.4 and 22.7% in GS_enr. Additionally, *Thermomonas*, *Paracoccus*, and *Roseisolibacter* were found in DS_enr with 7.8, 6.3, and 6.2% RA, respectively, and the *Ralstonia* genus was found in GS_enr with a RA of 8.6%. Only *Ignavibacterium* and *Thermomonas* were found in the starting sludges but with a RA < 0.2%.

Besides enriching for *Thiobacillus* and unclassified *Enterobacteriales*, the elemental sulfur incubations developed *Castellaniella* and *Paracoccus* for 12.7 and 4.6% RA in DS_enr and 14.2 and 14.3% in GS_enr, respectively. Furthermore, in the elemental sulfur assay, the genus *Thioprofundus* developed during the DS_enr with a RA of 12.6% and the *Citrobacter* genus was detected with a RA of 7.7% in GS_enr.

## Discussion

### Autotrophic Denitrification With Thiosulfate as Electron Donor

Analyzing the denitrification rates, thiosulfate-driven denitrification gave the highest rates among the SRCs tested for both enrichments (Table 3). The suitability of thiosulfate for this process is due to its high solubility and easy availability to the microorganisms (Di Capua et al., 2016). Thiosulfate has often been used in sulfur-based denitrification as starting substrate to enrich mixed inocula for autotrophic denitrifiers, although it was not the principal electron donor subject of the study (Krishnakumar and Manilal, 1999; Yang et al., 2018).

The five transfers on thiosulfate were completed within 22 and 20 days for DS and GS, respectively. The genus *Thiobacillus* comprises the microorganisms most commonly involved in sulfur-based denitrification (Zou et al., 2016), and it was thus expected to proliferate in the microbial community. In contrast, *Thiobacillus* sp. was only present in the DS ThEnr (Figure 3B). Species belonging to the *Sulfurimonas* genus were present in greater RA in GS ThEnr than DS ThEnr and most likely supported the faster denitrification activity in the GS ThEnr. Indeed, the *Sulfurimonas* genus is capable of using thiosulfate and other RSCs as electron donor for nitrate reduction (Takai et al., 2006). Moreover, in both enrichments, genera of heterotrophic denitrifiers were identified: members of the genus *Comamonas* were mostly present in the DS ThEnr, while *Microvirgula* genus members were detected in GS ThEnr. The presence of these genera suggests that heterotrophic activity was present in the enrichments, which was likely supported by endogenous or residual organics (Supplementary Table 1) as electron donor along the predominant autotrophic denitrification process. However, a 16S rRNA sequence analysis would be needed to better understand which microorganisms were active and actually contributed to the denitrification process (Blazewicz et al., 2013).

According to Eqs. 1–4, autotrophic denitrification with thiosulfate as electron donor produced more sulfate per mole of nitrate reduced (1.25 mol). Figure 2B shows that 1.12 and 1.05 mmol SO_4_^2–^/mmol NO_3_^–^ were produced, respectively, in DS ThEnr and GS ThEnr with a final sulfate concentration of 22.67 mM in both enrichments. Nitrate reduction, in accordance with what is reported by Oh et al. (2000), was not affected by sulfate that showed inhibitory effects only at concentrations exceeding 60 mM.

### Autotrophic Denitrification With Elemental Sulfur as Electron Donor

The six transfers of the S^0^Enr were carried out in 71 days for DS and 74 for GS. The enrichments were left inside the bottles between the 4th and 5th transfers for a longer time (19 days) as specified in *Section 2.3* since a slowdown in denitrification activity had been noted in the 3rd and 4th transfer. Despite the fact that denitrification rates are comparable to those obtained with the ThEnr (Table 3), S^0^Enr took three times longer to achieve stability in the denitrification rate. The denitrification rates obtained with DS S^0^Enr and GS S^0^Enr (Table 3) are 5.2 and 3.9 times faster than those found by Kostrytsia et al. (2018) who used 38.5 as molar ratio e^–^ donor/e^–^ acceptor with a temperature of 30°C and pH of 7.5. Kostrytsia et al. (2018) used autotrophic denitrifying biomass enriched for 3 months from an activated sludge collected from a denitrification tank of a municipal wastewater treatment plant. The main difference from their study is that the elemental sulfur source was supplied as particles with an average size between 2 and 4 mm, compared to 150 μm (100 mesh) in this study. The insoluble nature of elemental sulfur in water [solubility of the α-S_8_ at 20°C is only 5 μg L^–1^; Boulegue (1978)] and the larger size of the particles most likely slowed down the release and availability of electrons and consequently also the denitrification process. The increase in specific surface area obtained applying sulfur as powder (size of 100 mesh in the present study) helped in the formation of a biofilm increasing the contact between the microorganisms and the particles and consequently increasing the solubilization and bioavailability of elemental sulfur (Koenig and Liu, 2001).

The microbial community composition showed predominance of members of the genus *Thiobacillus* (Figure 3). Additionally, the genus *Parococcus* was present in the enrichment of both sludges. The genus *Parococcus* is a heterotrophic group, also able to use H_2_, sulfide, and thiosulfate as electron donor for the denitrification (Friedrich and Mitrenga, 1981). Thus, likely, elemental sulfur was converted to thiosulfate during its oxidation by nitrate, which is a suitable substrate for *Parococcus* (Friedrich and Mitrenga, 1981). Members of the genus *Thioprofundum* were also detected in DS S^0^Enr, which are microorganisms able to use elemental sulfur and other RSCs as electron donor for the reduction of nitrate (Takai et al., 2009).

In the elemental sulfur incubations, genera of heterotrophic denitrifiers were detected as well: *Castellaniella* in enrichments of both sludges and *Citrobacter* in GS S^0^Enr. As previously mentioned, TOC was below the detection limit in the S^0^Enr incubation (Supplementary Table 1), indicating the absence of organic compounds to be used as electron donor in heterotrophic denitrification. One hypothesis is that the biofilm, formed on the elemental sulfur surface, provides the endogenous organic compounds. Indeed, heterotrophic denitrifiers can remain active for long times (>100 days) in immobilized biomass systems, even when no external electron donor is provided (Costa et al., 2018). The activity of these bacteria might explain the gap between the measured and expected sulfate production (Figure 2). In fact, SO_4_^2–^ production was 0.6 mmol SO_4_^2–^/mmol NO_3_^–^ in DS S^0^Enr and 0.4 mmol SO_4_^2–^/mmol NO_3_^–^ in GS S^0^Enr, which is 30–50% less than expected from the stoichiometry (0.83 mmol SO_4_^2–^/mmol NO_3_^–^; Eq. 3). Thus, GS S^0^Enr enrichments show a greater RA of heterotrophic denitrifiers that do not contribute to the SO_4_^2–^ production since they do not use the elemental sulfur provided as electron donor. Besides, FTIR-ATR analyses on the solid material at the end of the incubation indicated a peak at 800–1800 cm^–1^ (data not shown), which is typical for ammonium sulfate precipitates (Kadam et al., 2011). These were not present on the solid particles at the beginning of the S^0^ enrichment (data not shown). Since ammonium was introduced with the mineral medium, this suggests that part of the sulfate had precipitated in the S^0^ incubations. Another hypothesis to explain the sulfate missing in the balance is the formation of sulfur intermediates that are not detected by the applied analytical method, e.g., polythionates (Miura and Watanabe, 2001).

### Autotrophic Denitrification With Pyrite as Electron Donor

Pyrite-driven autotrophic denitrification is an intricate process, involving not only the nitrogen and sulfur cycles, but also that of iron. The iron in pyrite is present in the ferrous form, which is oxidized during the process to ferric iron that binds with hydroxide ions and precipitates as Fe(OH)_3_ (Eq. 1). Moreover, Fe^2+^ and Fe^3+^ can also bind with other ions such as PO_4_^3–^ and SO_4_^2–^ (Li et al., 2016; Di Capua et al., 2020), or can precipitate again as FeS (Zhang et al., 2015).

Figure 2 shows that in the PyEnr, the sulfate concentration was always much lower than the theoretical values. Di Capua et al. (2020) also found a gap between the sulfate measured and sulfate theoretically expected, and analyzing the backwashing material of the recirculated pyrite-packed biofilter through FTIR-AR, sulfate sequestration due to its precipiation as iron sulfates such as Fe_2_(SO_4_)_3_⋅xH_2_O was observed. Another possible explanation for the missing sulfate is the involvement of sulfate-reducing bacteria (SRB) allowing the release of endogenous carbon and organics from the biofilm (Yang et al., 2017). Yang et al. (2017) found S in the form of polysulfide on the nanostructured pyrrhotite surface, indicating that, in their case, SRB were involved. In addition, Tong et al. (2017) found that other sulfur species than SO_4_^2–^, such as S_2_O_3_^2–^ and SO_3_^2–^, were present in the effluent of a pyrite upflow packed bed bioreactor. In the present study, no sulfur compound other than sulfate could be detected with the applied analytical methods. Furthermore, no SRB were detected in the enrichments (Figure 3B). FTIR-ATR analyses on the solid material withdrawn at the end of the transfers in PyEnr disclosed at peak at the 800–1800 cm^–1^ range (data not shown), indicating ammonium sulfate precipitation. Moreover, the pyrite sample also revealed a peak in the 2700–3700 cm^–1^ region (data not shown), which is characteristic of ferric sulfate precipitation (Majzlan et al., 2011; Di Capua et al., 2020). These results suggest sulfate precipitation onto the pyrite surface, thus leading to a lower sulfate concentration in solution.

Members of the genus *Ignavibacterium* were present in both enrichments with a major RA in GS PyEnr. *Ignavibacterium album*, the only representative of this genus, was isolated from a sulfide-rich hot spring (Lino et al., 2010) and it is able to oxidize sulfide and other RSCs (Liu et al., 2012). Sulfide in such genus may be intracellularly assimilated via cysteine synthase but its transport is poorly understood (Liu et al., 2012). The *I. album* genome does not encode enzymes that would allow it to use nitrate (Liu et al., 2012). Wang et al. (2015) found this genus in a system made for nitrate removal coupled to ferrous oxidation, while Zhang et al. (2015) associated it with elemental sulfur denitrification as well. Its role in such processes is not yet well studied. Some *Thiobacillus* species store elemental sulfur extracellularly as an intermediate in the oxidation pathway (Dahl and Prange, 2006; Berg et al., 2014). The autotrophic members of the denitrifying genus *Parococcus* (in DS PyEnr) and *Thioprofundum* (in DS PyEnr and GS PyEnr) were also found in the final enrichments and likely contributed to the autotrophic denitrification process as well.

### Autotrophic Denitrification With Sulfide as Electron Donor

The use of sulfide as electron donor for autotrophic denitrification is well known and has already been applied at full scale (Wu et al., 2016). Sulfide was also found to be a suitable substrate for the development of a selected community of specialized autotrophic, heterotrophic, and mixotrophic denitrifiers (Huang et al., 2021), where *Thiobacillus* was the enriched microorganisms with a maximum RA of 73.8% during the autotrophic phase.

With the complete oxidation of sulfide to sulfate, eight electrons are transferred for nitrate reduction, making this reaction one of the most energetically favorable processes (Di Capua et al., 2019). However, sulfide is well known to be inhibitory in too high concentrations for autotrophic denitrifiers (Cardoso et al., 2006) and in particular for *T. denitrificans* (Sublette et al., 1998). Sublette and Woolsey (1989) found that *T. denitrificans* was inhibited by sulfide concentrations as low as 0.1–0.2 mM. They isolated a sulfide-tolerant strain by exposing it to increasing sulfide concentrations up to 2.5 mM. Cardoso et al. (2006) instead found inhibition on a chemolithotrophic denitrifying enrichment at 7.5 mM sulfide.

In the present study, the sulfide concentration was kept at 3 mM from the first transfer with a pH of 7 (± 0.2) at which sulfide is 50% in HS^–^ form and 50% as H_2_S (Yongsiri et al., 2004). In the first three transfers, the denitrification was performed by a mix of autotrophic and heterotrophic activity since NO_3_^–^ was completely reduced even if sulfide (completely utilized) was provided at a substoichiometric concentration. The TOC, in fact, decreased from 975 (± 2.97) mg/L in DS and 1252 (± 9.39) mg/L in GS to under the detection limit along the three transfers (Supplementary Table 1). From the 4th enrichments onward, no denitrification activity could be detected anymore and no sulfide utilization was detectable. Likely, during the transfers, the applied sulfide concentration was not allowing the growth of the each time diluted denitrifying population.

In pyrite-driven denitrification, sulfide is released during pyrite solubilization. During this study, sulfide was never measurable in solution of the pyrite enrichments. The low pyrite solubilization likely did not allow an accumulation of sulfide in the bottles since it was immediately oxidized by the denitrification. This suggests that the use of pyrite is actually an approach in which sulfide is supplied at low, non-toxic concentrations, and released distributed over time.

### Choice of the Electron Donor and Practical Applications

This study showed that electron donor choice is an important factor for achieving high autotrophic denitrification rates and the selection of a RSC as a suitable substrate for practical applications should consider several aspects. It is convenient, if the wastewater already contains an eligible substrate, to use it directly as an electron donor. Otherwise, if the addition of an external electron donor is required, a cost-effective analysis is essential to optimize the process. Based on our results, the fastest electron donor for the autotrophic denitrification process was thiosulfate, considering the denitrification rates and the time required to reach a 100% denitrification efficiency. However, other facets must be considered as well to have a wider view of the entire process.

Wastewaters strongly contaminated with NO_3_^–^ require high thiosulfate concentrations for the denitrification, which will consequently lead to high effluent SO_4_^2–^ concentrations (Chung et al., 2014). Sulfate production is a parameter to evaluate for real applications since its concentration might surpass the 2000 European Union Directive on the drinking water legal limit of 250 mg/L (equal to 2.6 mM). The stoichiometry (Eqs. 1–3) indicates that SO_4_^2–^ production is in the order of S_2_O_3_^2–^ > S^0^ > FeS_2_ and Figure 2 confirms this difference, especially for pyrite-driven denitrification.

Thiosulfate is highly bioavailable for the microorganisms, meaning that in every transfer, its concentration had to be restored since it was quickly consumed. On the other hand, in the elemental sulfur and pyrite enrichments, the substrate was introduced only at the start of the first transfer. Their slow solubilization was enough to support the enrichments for all their transfers. Despite the several transfers of the elemental sulfur and pyrite particles, the solid substrate was still present and easily visible inside the bottles in the last transfer. This means that the full denitrification capacity was not yet achieved and there was thus no requirement to add more electron donor to the system. Considering the amount in mass of electron donors added in the present study in every experiment to treat this specific synthetic wastewater, and their prices (Table 4), thiosulfate results in the most expensive electron donor for real applications, while elemental sulfur is the cheapest (0.38 €/m^3^; Zhu and Getting, 2012) even if the latter was added in large excess and the stoichiometric molar ratio is for the benefit of thiosulfate. The reasons are mainly because (1) the price of thiosulfate per unit of mass is higher than that of elemental sulfur and (2) as thiosulfate is heavier than elemental sulfur (MW of S_2_O_3_^–^ and S^0^ are, respectively, 112.13 g/mol and 32.07 g/mol), more mass is needed. To reduce 1 mol nitrate (62 g), 0.625 mol thiosulfate is required (73 g) while 0.83 mol (27 g) of elemental sulfur is needed.

**TABLE 4:** Prices and cost estimates of the electron donors according to the utilization in the present study.

e^–^ donor	e^–^ donor added [kg/m^3^]	Price [€/kg e^–^ donor]	Cost [€/m^3^]
Thiosulfate	11.2*	0.17–0.21 ^a^	1.9–2.35
Elemental sulfur	2.56	0.15 ^b^	0.38
Pyrite	9.6	0.05–0.17 ^c^	0.48–1.63

*Calculated as overall of all the five transfers. ^*a*^Di Capua et al. (2019), ^*b*^Zhu and Getting (2012), ^*c*^Yang et al. (2017).

Pyrite’s price instead, as shown in Table 4, has a wide range, which makes the final cost of the process fluctuating, depending on the context of the application. Furthermore, pyrite-driven denitrification never reached 100% efficiency and further optimization of the system would be required before approaching full-scale application. Iron sulfide minerals have benefits such as stable pH and less by-product generation (SO_4_^2–^, NH_4_^+^, and N_2_O) that make it a practical option for engineering applications (Hu et al., 2020). Their purity is, however, a potential concern, as pyritic minerals often also contain traces of heavy metals, such as Cu or As.

Acetic acid (CH_3_COOH) and methanol (CH_3_OH) are two of the most commonly used organic electron donors for heterotrophic denitrification (Eqs. 5 and 6) (Mohseni-Bandpi et al., 2013):

(5) 5CH3COOH+8NO3→-4N2+10CO2+6H2O+8OH-

(6) 5CH3OH+6NO3→-3N2+5CO2+7H2O+6OH-

Table 5 shows the estimated costs to treat the specific synthetic wastewater used in this study with RSCs (Eqs. 1–3) as well as acetic acid and methanol according to their stoichiometry (Eqs. 5 and 6). The two organic electron donors have higher prices than the inorganic ones, especially acetic acid, which is between 7 and 30 times more expensive than the tested RSCs. Nevertheless, the mass of thiosulfate required per m^3^ of wastewater treated makes its final cost similar to that of methanol. However, comparing the costs of the organic electron donors deduced stoichiometrically (Table 5) with that of elemental sulfur actually added in the present study (Table 4) shows that they were comparable for methanol, but elemental sulfur was 2.5 times cheaper than acetic acid.

**TABLE 5:** Prices and cost estimates of the Sulfurous electron donors used in the present study and the most common heterotrophic electron donors according to stoichiometry.

e^–^ donor	e^–^ donor theoretically required [kg/m^3^]	Price [€/kg e^–^ donor]	Cost [€/m^3^]
Thiosulfate	1.4	0.17–0.21 ^a^	0.24–0.29
Elemental sulfur	0.53	0.15 ^b^	0.08
Pyrite	0.8	0.05–0.17 ^c^	0.04–0.14
Acetic acid	0.63	1.5 ^a^	0.94
Methanol	0.53	0.48–0.61 ^a^	0.29–0.33

*^*a*^Di Capua et al. (2019), ^*b*^Zhu and Getting (2012), ^*c*^Yang et al. (2017).*

## Conclusion

A selected community of autotrophic denitrifiers was developed, for the first time, using pyrite as electron donor. The presence of the species *I. album*, especially in the pyrite enrichment with GS sludge, deserves further studies to better understand its role in the process. Thiosulfate supported the highest denitrification rates, 1.5 and 6 times faster than elemental sulfur and pyrite, respectively. Moreover, it was the electron donor that responded better to the enrichment by increasing the denitrification rate along the enrichment. The analysis on the tested electron donors for application in nitrogen removal processes revealed elemental sulfur as the most promising electron donor for the denitrification of the synthetic wastewater investigated.

## Data Availability Statement

The datasets presented in this study can be found in online repositories. The names of the repository/repositories and accession number(s) can be found below: https://www.ncbi.nlm.nih.gov/bioproject/707573, PRJNA707573.

## Author Contributions

MC: conceptualization, data curation, investigation, methodology, formal analysis, writing—original draft, writing—review and editing, and visualization. AF: conceptualization, supervision, and writing—review and editing. RC: conceptualization, supervision, and writing—review and editing. XZ: supervision and writing—review and editing. PL: supervision, project administration, writing—review and editing, and funding acquisition. All authors contributed to the article and approved the submitted version.

## Conflict of Interest

The authors declare that the research was conducted in the absence of any commercial or financial relationships that could be construed as a potential conflict of interest.

## Abbreviations

DS: Digested sludge from a wastewater treatment plant of the dairy industry
DS_enr: Last transfer of the enrichment with DS
GS: Granular sludge from a dairy wastewater treatment plant
GS_enr: Last transfer of the enrichment with GS
MW: Molecular weight
OTU: Operational taxonomic units
PyEnr: Enrichment with pyrite as electron donor
RA: Relative abundance
rpm: Revolutions per minute
RSC: Reduced sulfur compound
S^0^Enr: Enrichment with elemental sulfur as electron donor
S^2–^Enr: Enrichment with sulfide as electron donor
SRB: Sulfate-reducing bacteria
ThEnr: Enrichment with thiosulfate as electron donor
TOC: Total organic carbon
TS: Total solids
UASB: Upflow anaerobic sludge blanket
VS: Volatile solids.
