# Supplementary material for: Enrichment of Autotrophic Denitrifiers From Anaerobic Sludge Using Sulfurous Electron Donors
Source: Front Microbiol. 2021 Jun 7;12:678323. doi: 10.3389/fmicb.2021.678323 (PMC8215349; doi:10.3389/fmicb.2021.678323)
Supplement: Supplementary file 1 [file Data_Sheet_1.PDF]

Supplementary Material

Supplementary Figures

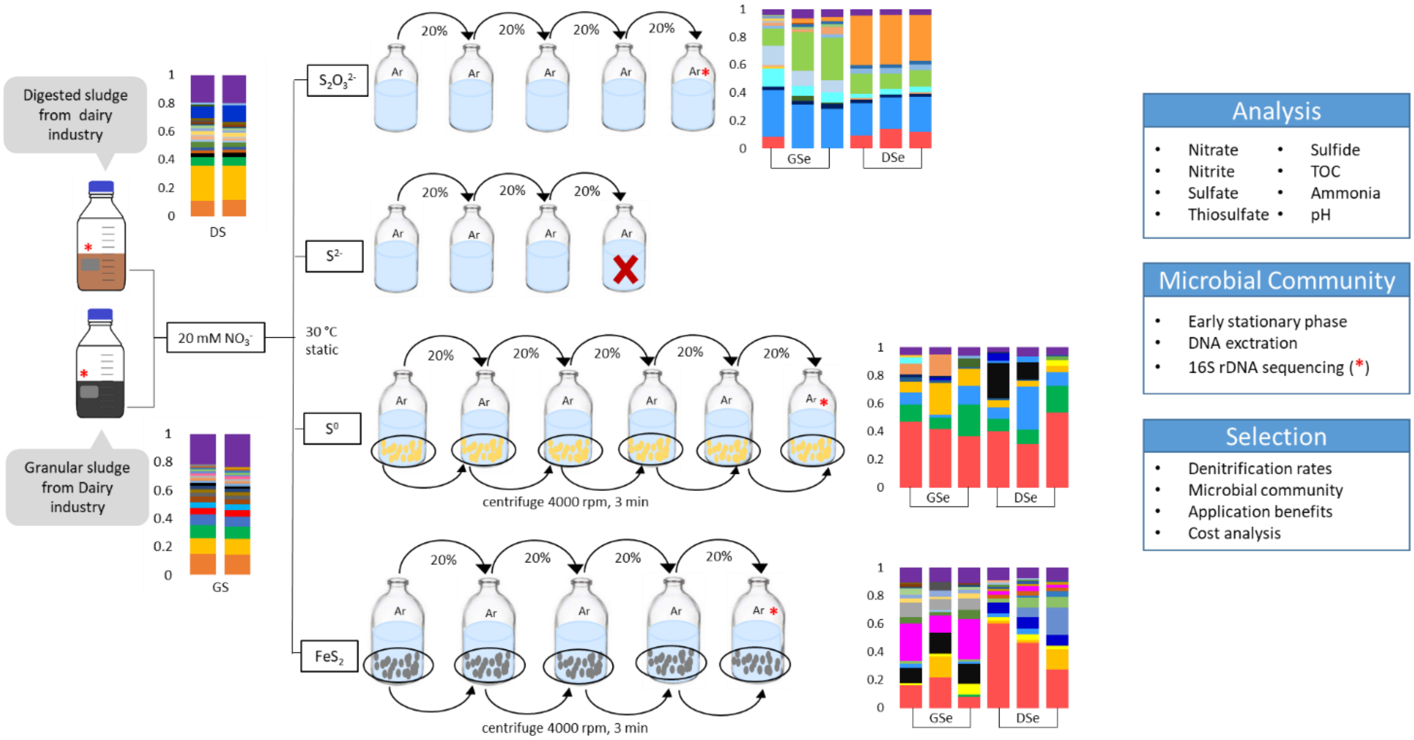

Supplementary Figure 1. Set up configuration adopted for the experimental work. The red asterisk indicates the step in which samples were taken for 16s rDNA sequencing analysis.

Supplementary Tables

Supplementary Table 1. TOC (mg/L) present at the beginning of every transfer for all the electron donors and both sludges (DS and GS)

	1st transfer	*2nd transfer*	*3rd transfer - last transfer*
**Thiosulfate_DS**	753.68 ± 11.35	113.67 ± 4.64	< 5
**Thiosulfate_GS**	945.45 ± 14.91	98.27 ± 29.35	< 5
**Sulfate_ DS**	975 ± 2.97	325 ± 11.95	< 5
**Sulfate_ GS**	1252 ± 9.39	417.5 ± 3.05	< 5
**Elemental sulfur_ DS**	886 ± 11.21	143 ± 1.38	< 5
**Elemental sulfur_ GS**	991.65 ± 16.01	301 ± 4.98	< 5
**Pyrite_ DS**	912 ± 1.51	120 ± 1.22	< 5
**Pyrite_ GS**	1158 ± 1.02	171.6 ± 8.6	< 5
